# Data on proteomic profiling of cells and extracellular vesicles of the melittin-resistant *Acholeplasma laidlawii* strain

**DOI:** 10.1016/j.dib.2019.104169

**Published:** 2019-06-21

**Authors:** E.S. Medvedeva, A.A. Mouzykantov, N.B. Baranova, M.A. Dramchini, O.A. Chernova, V.M. Chernov

**Affiliations:** a Kazan Institute of Biochemistry and Biophysics, FRC Kazan Scientific Center of RAS, Russia; b Kazan (Volga Region) Federal University, Russia

**Keywords:** *Acholeplasma laidlawii*, Melittin, Susceptibility, Extracellular vesicles, Proteome, 2DE, MALDI-TOF/TOF MS, LC–ESI-MS/MS

## Abstract

*Acholeplasma laidlawii* (class Mollicutes), a major contaminant of cell cultures, quickly adapts to various classes of antimicrobials, including antimicrobial peptides. The extracellular vesicles of this bacterium can play a significant role in the development of drug-resistance Chernov et al., 2018. We compared the cellular and vesicular proteomes of *A. laidlawii* strains with differing susceptibility to melittin (an antimicrobial peptide from bee venom), the genomes of which we have previously sequenced. We extracted soluble proteins from cells and extracellular vesicles of the *A. laidlawii* PG8R_Mel_ strain showing an increased resistance to melittin, and compared them with the cellular proteome and a previously obtained vesicular proteome of the original (reference) *A. laidlawii* PG8B strain Chernov et al., 2014. The cellular proteome profile of the *A. laidlawii* strains differing in susceptibility to melittin was determined by using two-dimensional gel electrophoresis and MALDI-TOF/TOF MS. Here we present the cellular proteins that were differentially expressed. The vesicular proteome profile was determined by using one-dimensional electrophoresis and chromatography-mass spectrometry. A list of the extracellular vesicles proteins of the melittin-resistant *A. laidlawii* strain is presented here.

**Table:** Specifications Table

Subject area	*Biology*
More specific subject area	*Mollicute proteomics; vesiculome*
Type of data	*Table Excel*
How data was acquired	*2-dimensional gel electrophoresis, MALDI-TOF/TOF MS, 1D SDS-PAGE, LC–ESI-MS/MS*
Data format	*Filtered, analyzed*
Experimental factors	*A. laidlawiistrains with differential susceptibility to melittin*
Experimental features	*The separation of cellular proteins of A. laidlawii strains was carried out using two-dimensional electrophoresis with subsequent identification of the obtained polypeptides by mass spectrometry. The differential expression of proteins was quantified by the PDQuest. The vesicles were obtained by ultracentrifugation; their purity was validated by PCR and TEM. Proteome profiling of vesicular proteins was performed using 1D-LC-ESI-MS/MS.*
Data source location	*KIBB FRC Kazan Scientific Center of RAS, Kazan, Russia*
Data accessibility	*The data are available with this article*
Related research article	*V.M. Chernov, A.A. Mouzykantov, N.B. Baranova, E.S. Medvedeva, T.Y. Grygorieva, M.V. Trushin, I.E. Vishnyakov, A.V. Sabantsev, S.N. Borchsenius, O.A. Chernova, Extracellular membrane vesicles secreted by mycoplasma Acholeplasma laidlawii PG8 are enriched in virulence proteins, J. Proteomics 110 (2014) 117–128.*https://doi.org/10.1016/j.jprot.2014.07.020*.*

**Table:** 

**Value of the data** • These data are the first that document the changes in the expression of cellular proteins of *A. laidlawii* strains differing in susceptibility to melittin. • These data are the first that document proteins identified in the extracellular vesicles of melittin-resistant *A. laidlawii* PG8R_Mel_. • These data may be of value for deep annotation of *A. laidlawii* proteome in terms of post-translational modifications. • These data may be useful for elucidating the molecular mechanisms of adaptation of the smallest fast evolving prokaryotes to antimicrobial peptides, and for the role of their extracellular vesicles in this process.

## 1 Data

**Fig. 1:**
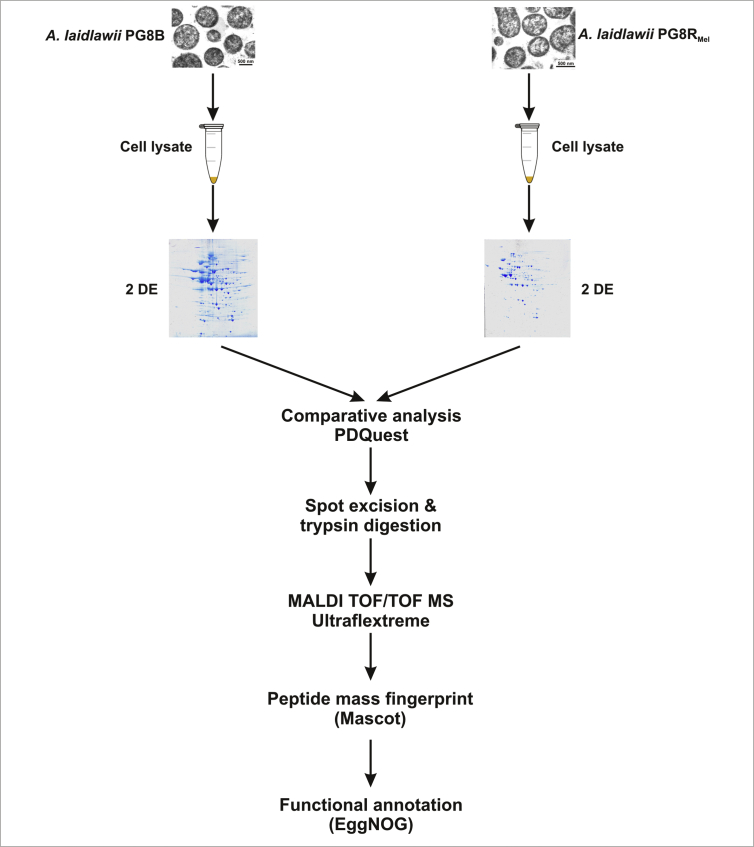
The experimental design used to compare the proteome of cells of the *A. laidlawii* strains that differ in their susceptibility to melittin.

**Fig. 2:**
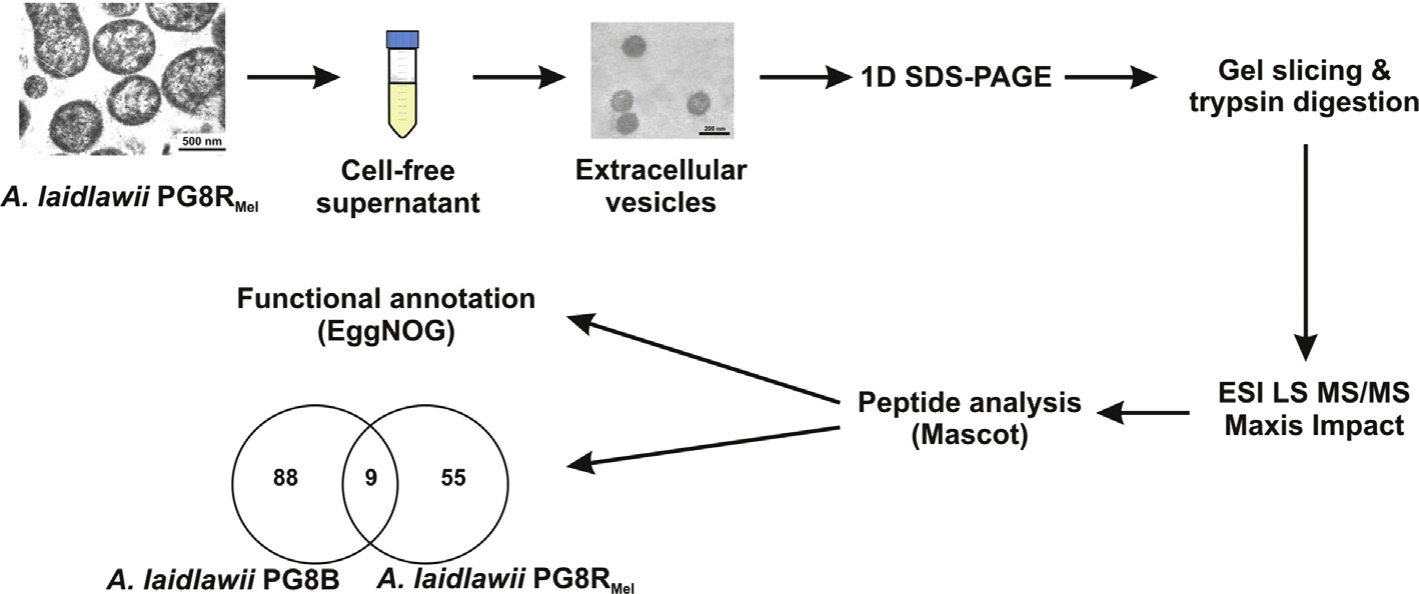
The experimental design that was used to derive the proteome of the extracellular vesicles produced by the melittin-resistant *A. laidlawii* PG8R_Mel_ strain.

The data document differentially expressed cellular proteins in two *A. laidlawii* strains – the reference *A. laidlawii* PG8B and the melittin-resistant *A. laidlawii* PG8R_Mel_ derived from it, as well as the protein profile of extracellular vesicles derived from *A. laidlawii* PG8R_Mel_ (Fig. 1, Fig. 2). The cell proteins were separated by two-dimensional gel electrophoresis and the differences in protein profiles of the *A. laidlawii* strains were analyzed using PDQuest software. Mass spectrometry revealed that there were 28 protein spots that were differentially expressed; three biological replicates were analyzed. The list of the proteins identified as differentially expressed in these strains as well as the information about their gene ontology (functional annotation) are presented in Supplementary Table 1. The qualitative composition of vesicular proteins was determined using 1D SDS-PAGE and LC-ESI-MS-MS. A list of the 64 identified vesicular proteins and their functional classification according to EggNOG are presented in Supplementary Table 2. The *A. laidlawii* PG8R_Mel_ EVs contain the proteases (ACL_RS02635, ACL_RS03650, ACL_RS04935, ACL_RS05845) which may have a role in degrading the antimicrobial peptides and the development of drug-resistance [1]. A list of the specific proteins derived from the *A. laidlawii* PG8R_Mel_ vesicles compared to those in *A. laidlawii* PG8B is presented in Supplementary Table 3.

## 2 Experimental design, materials, and methods

### 2.1 Bacterial strains and culture conditions

The *A. laidlawii* cells were grown in a modified liquid Edward's medium (EM) at 37 °C [2]. Melittin is an antimicrobial peptide from bee venom. A resistant strain of *A. laidlawii* (PG8R_Mel_) strain was grown in EM at 37 °C containing 15 μg ml^−1^ melittin. For the proteome analysis, *A. laidlawii* PG8R_Mel_ strain was grown for one passage without melittin.

### 2.2 Proteins preparation

Proteins from cells of *A. laidlawii* were isolated as described previously [3]. The *A. laidlawii* cells were collected by centrifugation at 6000*g* for 20 min. The pellet was washed twice with buffer (150 mM NaCl, 50 mM Tris, 2 mM MgCl_2_.6H_2_O, pH 7.4) and once in the same buffer with protease inhibitor PMSF (Fluka, Germany). All procedures were performed at + 4 °C. Then the cells were frozen in liquid nitrogen and stored at −84 °C. The pellet of cells was treated with CHAPS and a mixture of nucleases Micrococcal Nuclease Mix (Thermo Fisher Scientific, USA). The resulting proteins were dissolved in a solution containing 8 M urea, 2 M thiourea, 5% ampholines (pH 3–10), 80 mM dithiothreitol (DTT), 5% CHAPS and 1.67% NP 40. The protein concentration in the samples was measured by the Bradford method using the Quick Start Bradford Dye Reagent (Bio-Rad Laboratories, Inc., USA).

### 2.3 2D-PAGE and gel analysis

Proteins were separated using 2DE as described previously [4]. Isoelectrofocusing was performed in glass tubes in 4% polyacrylamide gel (water Milli-Q, 8 M urea, 4% acrylamide/bis-acrylamide, 1.75% ampholines (pH 3–10), 3.5% ampholines (pH 5–8), 1.8% CHAPS and 0.6% NP-40, 0.1% TEMED, 0.02% ammonium persulfate). Isoelectrofocusing was done in the following regime: 100 V–200 V–300 V–400 V–500 V–600 V — for 45 min, 700 V — for 10 h, 900 V — for 2 h. After isoelectrofocusing, tubes were equilibrated for 30 min in a buffer containing 6 M urea, 30% glycerol, 62.5 mM Tris–HCl (pH 6.8), 2% SDS, bromophenol blue and 20 mM DTT. Then the tubes were placed onto surface of 12% polyacrylamide gel and fixed in place with 0.9% agarose containing bromophenol blue. Electrophoresis was performed in Tris-glycine buffer with cooling at the following regime: 40 mA — for 20 min, 80 mA — for 2 h, 70 mA — for 2.5 h. The gels were stained with Coomassie Brilliant Blue (CBB) G-250. The gels were scanned and analyzed with PDQuest (version 8.0.1) software (Bio-Rad Laboratories, Inc., USA). Spots that were present in all three replicates were selected for subsequent comparison and identification. A cutoff value was set at a 1.5-fold increase or decrease.

### 2.4 Protein trypsinolysis

Proteins were extracted from the gel and hydrolyzed using the protocol described in Ref. [5]. The protein spots were cut out from the gel and washed in ddiH_2_O (15 min) and acetonitrile: 200 mM NH_4_HCO_3_ (1:1) at 50°С 30 min. Protein reduction was performed using 10 mM DTT 100 мМ NH_4_HCO_3_ for 1 h, followed by alkylation using a mixture of 50 mM iodoacetamide and 100 mM NH_4_HCO_3_ in the dark for 45 min at room temperature. The gels were incubated in acetonitrile, dried and incubated in trypsin Gold (Promega, USA) solution for 60 min at 4 °C. Trypsinolysis was performed at 37 °C overnight. To extract peptides, 20 μl of a solution containing 0.1% (v/v) trifluoroacetic acid (TFA) in deionized water was added to gel fragments that were then incubated in an ultrasonic bath for 10 min. The resulting supernatants were sampled into separate tubes. A second extraction was carried out by adding, 20 μl of a 0.1% (v/v) solution of TFA in 50% acetonitrile was added, and then samples were incubated in an ultrasonic bath for 10 min. Samples were analyzed with mass-spectrometry.

### 2.5 Protein identification by MALDI TOF∖TOF MS

The identification of differentially expressed proteins was performed using a MALDI-TOF/TOF mass-spectrometer Ultraflex III BRUKER (USA) with a UV-laser in the positive ion mode in the diapason of 500–4000 Da using reflectron [3]. The accuracy of the measured monoisotopic masses in the reflect-mode after calibration to the peaks of trypsin autolysis was 0.007%; possible methionine oxidation by atmospheric oxygen and modification of cysteines by acrylamide were taken into consideration. The proteins were identified from the masses of proteolytic fragments using Mascot Peptide Fingerprint (Matrix Science, USA) software and NCBI database containing the complete genome of *A. laidlawii* PG-8A. A protein score of >44 was considered a significant matched (p < 0.05).

### 2.6 Extraction and purification of extracellular vesicles

The isolation of the *A. laidlawii* PG8R_Mel_ extracellular vesicles was performed according to Ref. [2]. The cells were pelleted by centrifugation at 6000*g* for 20 min. The supernatant was filtered through a 0.1 μm Minisart High Flow Syringe Filter (Sartorius, Germany), and the filtrate was concentrated 20-fold using concentrator Vivacell 100 (Sartorius, Germany). The vesicles were collected by ultracentrifugation (100,000 *g*, 1 h, 8 °C) (Beckman Coulter Optima™ MAX-E). The pellet was suspended in buffer (50 mM Tris-HCl, pH = 7.4; 150 mM NaCl; 2 mM MgCl_2_). The suspension was placed on a stepwise density gradient 20%–40% Optiprep (Sigma, USA) and ultracentrifuged. The vesicular fraction was collected, diluted threefold in buffer and then ultracentrifuged again. The pellet was resuspended in buffer supplemented with 1 mM PMSF (Fluka, Germany) and stored at 8 °C. The absence of microbial cells in the vesicle preparation was tested using TEM, plating on EM and PCR analysis with primers for marker nucleotide sequences [2].

### 2.7 SDS-PAGE and trypsinolysis

Preparation of vesicular proteins, their separation using 1D SDS-PAGE and trypsinolysis were performed according to Ref. [2]. The proteins from purified EVs were dissolved in a buffer containing 25 mM Tris-HCl (pH 6.8), 5% glycerol, 0.05% bromophenol blue, 1% SDS and 50 mM DTT. Proteins were separated using 1D SDS-PAGE (12% resolving gel). The gel was subsequently stained with CBB G-250. The protein fractions were cut out from the gel and washed for 15 min in ddiH_2_O and then in an solution containing a 1:1 mixture of acetonitrile and 200 mM NH_4_HCO_3_ at 50°С for 30 min. Protein reduction was performed using a solution containing 10 mM DTT and 100 мМ NH_4_HCO_3_ for 1 h, followed by alkylation using in a solution of 50 mM iodoacetamide and 100 mM NH_4_HCO_3_ in the dark for 45 min at room temperature. The gels were incubated in acetonitrile, dried and incubated in trypsin Gold (Promega, USA) solution for 60 min at 4 °C. The trypsinolysis was performed overnight at 37 °C. To extract the peptides from the gel, 20 μl of a 0.5% TFA solution was added to each tube and the tubes incubated in an ultrasonic bath for 10 min. The extract was transferred to a clean tube and dried in a centrifugal evaporator. Samples were analyzed with mass-spectrometry.

### 2.8 LC–ESI-MS/MS analysis

LC–ESI-MS/MS analysis and identification of proteins was as described previously [2]. The trypsinolized samples were dissolved in a mixture of 98.9% water, 1% methanol, 0.1% formic acid (v/v), loaded to an Acclaim PepMap RSLC column (Thermo Fisher Scientific, USA) and eluted for five h, increasing the concentration of a mixture of 99.9% acetonitrile and 0.1% formic acid (v/v) from 2 to 60%. Mass spectra were obtained on a mass spectrometer Maxis Impact (Bruker, Germany) equipped with a HPLC system Dionex Ultimate 3000 Series (Thermo Fisher Scientific, USA). The MS1 mass spectra were obtained as follows: detection of molecular ions was performed in the range was 300–2000 *m*/*z*, with a signal accumulation time of 250 ms. To obtain MS2 spectra, ions with a signal-to-noise ratio of at least 400 and a charge from 2 to 5 were selected. Ion detection was performed in the range of 200–2000 *m*/*z*, with a signal accumulation time of 50 ms for each parent ion. The measurement accuracy was 0.6 Da. The resulting MS/MS spectra were analyzed using the MASCOT program (Matrix Science, Inc.). Protein identification was considered reliable when at least two peptides with different amino acid sequences with a pepscore value ≥ 15 were detected.

### 2.9 Analysis of amino acid sequences *in silico*

BLAST and SwissProt were used to analyze the amino acid sequences of the domains in the proteins [6]. The functions of each protein were classified according to EggNOG [7].

## References

[1] Chernov V.M., Chernova O.A., Mouzykantov A.A., Medvedeva E.S., Baranova N.B., Malygina T.Y., Aminov R.I., Trushin M.V. (2018). Antimicrobial resistance in mollicutes: known and newly emerging mechanisms. FEMS Microbiol. Lett..

[2] Chernov V.M., Mouzykantov A.A., Baranova N.B., Medvedeva E.S., Grygorieva T.Y., Trushin M.V., Vishnyakov I.E., Sabantsev A.V., Borchsenius S.N., Chernova O.A. (2014). Extracellular membrane vesicles secreted by mycoplasma *Acholeplasma laidlawii* PG8 are enriched in virulence proteins. J. Proteomics.

[3] Chernov V.M., Chernova O.A., Medvedeva E.S., Mouzykantov A.A., Ponomareva A.A., Shaymardanova G.F., Gorshkov O.V., Trushin M.V. (2011). Unadapted and adapted to starvation *Acholeplasma laidlawii* cells induce different responses of *Oryza sativa*, as determined by proteome analysis. J. Proteomics.

[4] Görg A., Obermaier C., Boguth G., Harder A., Scheibe B., Wildgruber R., Weiss W. (2000). The current state of two-dimensional electrophoresis with immobilized pH gradients. Electrophoresis.

[5] Shevchenko A., Wilm M., Vorm O., Mann M. (1996). Mass spectrometric sequencing of proteins from silver-stained polyacrylamide gels. Anal. Chem..

[6] UniProt Consortium (2019). UniProt: a worldwide hub of protein knowledge. Nucleic Acids Res..

[7] Huerta-Cepas J., Szklarczyk D., Forslund K., Cook H., Heller D., Walter M.C., Rattei T., Mende D.R., Sunagawa S., Kuhn M., Jensen L.J., von Mering C., Bork P. (2016). eggNOG 4.5: a hierarchical orthology framework with improved functional annotations for eukaryotic, prokaryotic and viral sequences. Nucl. Acids Res..

